# The prognostic value of histological subtype in patients with metastatic bladder cancer

**DOI:** 10.18632/oncotarget.16083

**Published:** 2017-03-10

**Authors:** Cheng Chen, Linkun Hu, Ye Chen, Jianquan Hou

**Affiliations:** ^1^ Department of Urology, the First Affiliated Hospital of Soochow University, Suzhou, People's Republic of China

**Keywords:** urinary bladder neoplasms, histology, distant metastasis, prognosis, survival

## Abstract

We aim to evaluate the prognostic effect of the histological sub-type in patients with metastatic bladder cancer based on the Surveillance Epidemiology and End Results database. A total of 2634 eligible patients were included. The histological subtypes were: transitional cell carcinoma (TCC; 75.2%); adenocarcinoma (3.3%); squamous cell carcinoma (SQCC; 4.1%); and small cell carcinoma (4.3%). A significant association of adenocarcinoma with better survival outcomes (*P* < 0.015), and that of SQCC with worse outcomes (*P* < 0.001) was observed. On multivariate analysis, adenocarcinoma was significantly associated with longer and SQCC with shorter survival time as compared to TCC. Overall, 1331 (50.5%) patients had a single metastatic site and 523 (19.9%) had multiple sites involved. Single-site metastasis had a better survival outcome than multiple metastases (*P* < 0.001). Histological sub-type and presence of multiple metastatic sites are independent predictors of survival time. Prospective, in-depth research is needed to determine optimal therapeutic strategies for different histological subtypes of bladder cancer with different metastatic patterns.

## INTRODUCTION

Bladder cancer (BC) has the fifth highest incidence among all cancers in the United States; an estimated 76960 new cases of BC and 16390 deaths from BC occurred in 2016 [1]. It is also the 9th most common cancer and the 13th most common cause of death worldwide [2]. Urothelial carcinoma (transitional cell carcinoma, TCC) is the most common histological sub-type of primary BC, which accounts for > 90% of all bladder tumors. Other histological subtypes include squamous cell carcinoma (SQCC), adenocarcinoma (AD), small cell carcinoma (SCC) and some other rare non-epithelial subtypes. Among these, SQCC, AD and SCC account for less than 5%, 2% [3] and 1% [4, 5] of all primary bladder tumors, respectively.

The rare occurrence of non-TCC subtypes precludes large, prospective studies to compare outcomes. Retrospective and cohort analyses remain the primary tools for clinical research. Previous studies of the impact of non-TCC histology on survival have yielded inconsistent results [6–9]. Some have reported no difference in survival between SQCC and TCC [7–9]. However, a multi-institutional retrospective study to assess the impact of non-TCC histology on bladder cancer-specific outcomes in patients treated with radical cystectomy [6] suggested that non-TCC histology portends worse clinical outcomes. This may reflect a greater propensity for locally aggressive disease and distant metastasis, as well as differential response to chemotherapy or radiotherapy.

Approximately 25% of all TCC patients will have muscle-invasive and metastatic disease. The prevalence of bone metastasis was reported to be 30–40% in patients with metastatic TCC [10] and the presence of distant (lung, liver, bone) metastases were reported to correlate with shortened survival [11–17]. However, the effect of other histological subtypes on clinical outcomes in patients with metastatic BC remains unclear. Thus, we evaluated TCC and three other rare BC histological sub-types in a contemporary, population-based cohort with an emphasis on the metastatic frequency of BC histology and their effect on overall- and cancer-specific survival.

## RESULTS

Data pertaining to 2634 patients who had stage M1 BC, and information on specific metastatic site in the period between January 2010 and December 2013, were extracted. The baseline demographic and clinical characteristics are summarized in Table 1. Of all patients with metastatic BC, the histological subtypes included TCC (*n* = 1981 [75.2%]), AD (*n* = 88 [3.3%]), SQCC (*n* = 107 [4.1%]) and SCC (*n* = 114 [4.3%]). A total of 1331 (50.5%) patients had a single metastatic site (bone, brain, liver or lung), while 523 (19.9%) had metastatic lesions at more than one site. In single metastatic BC patients, 575 (43.2%) of them had bone metastasis, 24 (1.8%) with brain metastasis, 280 (21%) with liver metastasis and 452 (34%) with lung metastasis.

**Table 1:** Clinical features associated with various histological subtypes of bladder cancer patients

Variables	Overall Patients	Transitional cell carcinoma	Adenocarcinoma	Squamous cell carcinoma	Small cell carcinoma	*P* value
No. of Patients	2634					
Age (*n*(%))						**< 0.001**
≤ 65	836 (31.7%)	615 (31%)	47 (53.4%)	40 (37.4%)	31 (27.2%)	
> 65	1798 (68.3%)	1366 (69%)	41 (46.6%)	67 (62.6%)	83 (72.8%)	
Gender (*n*(%))						**< 0.001**
male	1853 (70.3%)	1418 (71.6%)	58 (65.9%)	52 (48.6%)	94 (82.5%)	
female	781 (29.7%)	563 (28.4%)	30 (34.1%)	55 (51.4%)	20 (17.5%)	
Race (*n*(%))						**0.003**
White	2227 (84.5%)	1692 (85.5%)	65 (73.9%)	85 (79.4%)	105 (92.1%)	
Black	262 (9.9%)	185 (9.4%)	18 (20.5%)	15 (14%)	7 (6.1%)	
Other	142 (5.4%)	101 (5.1%)	5 (5.7%)	7 (6.5%)	2 (1.8%)	
Unknown	3 (0.1%)					
Marital status (*n*(%))						**0.001**
Single	426 (16.2%)	307 (16.2%)	27 (30.7%)	22 (21.8%)	8 (7.2%)	
Married	1262 (47.9%)	963 (51%)	42 (47.7%)	50 (49.5%)	71 (64%)	
Divorced/Separated	338 (12.8%)	260 (13.8%)	7 (8%)	9 (8.9%)	15 (13.5%)	
Widowed	491 (18.6%)	360 (19%)	12 (13.6%)	20 (19.8%)	17 (15.3%)	
Unknown	117 (4.4%)					
T stage (*n*(%))						**< 0.001**
T0 + Tx	585 (22.2%)	322 (16.3%)	28 (31.8%)	21 (19.6%)	22 (19.3%)	
T1	385 (14.6%)	316 (16%)	10 (11.4%)	11 (10.3%)	17 (14.9%)	
T2	948 (36%)	813 (41%)	17 (19.3%)	25 (23.4%)	44 (38.6%)	
T3	227 (8.6%)	177 (8.9%)	13 (14.8%)	7 (6.5%)	10 (8.8%)	
T4	489 (18.6%)	353 (17.8%)	20 (22.7%)	43 (40.2%)	21 (18.4%)	
N stage (*n*(%))						0.008
N0 + Nx	1815 (68.9%)	1335 (67.4%)	71 (80.7%)	64 (59.8%)	70 (61.4%)	
N1	245 (9.3%)	189 (9.5%)	6 (6.8%)	18 (16.8%)	11 (9.6%)	
N2	439 (16.7%)	344 (17.4%)	11 (12.5%)	17 (15.9%)	29 (25.4%)	
N3	135 (5.1%)	113 (5.7%)	0 (0%)	8 (7.5%)	4 (3.5%)	
Grade (*n*(%))						**< 0.001**
G1	21 (0.8%)	12 (0.8%)	1 (2%)	7 (9.2%)	0 (0%)	
G2	80 (3%)	49 (3.1%)	9 (18.4%)	20 (26.3%)	0 (0%)	
G3	657 (24.9%)	481 (30.3%)	36 (73.5%)	37 (48.7%)	31 (50.8%)	
G4	1126 (42.7%)	1045 (65.8%)	3 (6.1%)	12 (15.8%)	30 (49.2%)	
Unknown	750 (28.5%)					
Surgery (*n*(%))						**< 0.001**
No	845 (32.1%)	484 (24.5%)	41 (46.6%)	42 (39.3%)	27 (23.7%)	
Yes	1784 (67.7%)	1494 (75.5%)	47 (53.4%)	65 (60.7%)	87 (76.3%)	
Unknown	5 (0.2%)	3 (0.2%)	0 (0%)	0 (0%)	0 (0%)	
Radiotherapy (*n*(%))						0.414
No	2055 (78%)	1521 (77.5%)	72 (81.8%)	87 (82.9%)	86 (75.4%)	
Yes	553 (21%)	442 (22.5%)	16 (18.2%)	18 (17.1%)	28 (24.6%)	
Unknown	26 (1%)					
Distant metastasis (*n*(%))						**0.031**
Single site (*n*(%))	1331 (50.5%)	1014 (51.2%)	41 (46.6%)	44 (41.1%)	54 (47.4%)	
Only bone	575 (43.2%)	468 (46.2%)	17 (43.6%)	15 (34.1%)	22 (40.7%)	0.358
Only brain	24 (1.8%)	20 (2%)	0 (0%)	0 (0%)	1 (1.9%)	0.635
Only liver	280 (21%)	179 (17.7%)	10 (24.4%)	5 (11.4%)	29 (53.7%)	**< 0.001**
Only lung	452 (34%)	347 (34.2%)	14 (34.1%)	24 (54.5%)	2 (3.7%)	**< 0.001**
Multiple sites	523 (19.9%)	377 (27.1%)	15 (26.8%)	15 (25.4%)	38 (41.3%)	
Unknown	780 (29.6%)					
Histology (*n*(%))						
Transitional cell carcinoma	1981 (75.2%)					
Adenocarcinoma	88 (3.3%)					
Squamous cell carcinoma	107 (4.1%)					
Small cell carcinoma	114 (4.3%)					
Other	462 (17.5%)					

**Figure 1:**
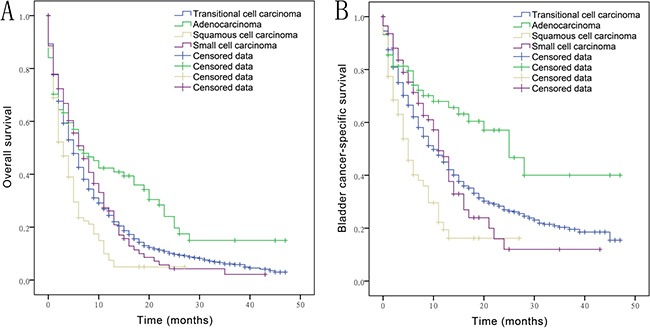
Overall survival (**A**) and bladder cancer-specific survival (**B**) in bladder cancer patients with transitional cell carcinoma, adenocarcinoma, squamous cell carcinoma, small cell carcinoma with distant metastasis.

Median duration of follow-up was 5 months (range, 0 to 47). A total of 2134 (81%) patients died in the group. Kaplan-Meier curves and median OS and CSS were calculated. The median OS and CSS for TCC were 5 and 10 months (range, 0 to 47), respectively; 6 and 25 months (range, 0–47) for AD, 3 and 5 months (range, 0–27) for SQCC and 7 and 11 months (range, 0–43) for SCC, respectively. As shown in Figure 1, the median OS and CSS for AD was significantly higher than that for other histological types (*P* < 0.015). The median OS and CSS for SQCC were significantly lower than that for the other histological types (*P* < 0.001). However, no significant difference in median OS and CSS was observed between TCC and SCC (*P* = 0.877 and 0.686, respectively).

**Figure 2:**
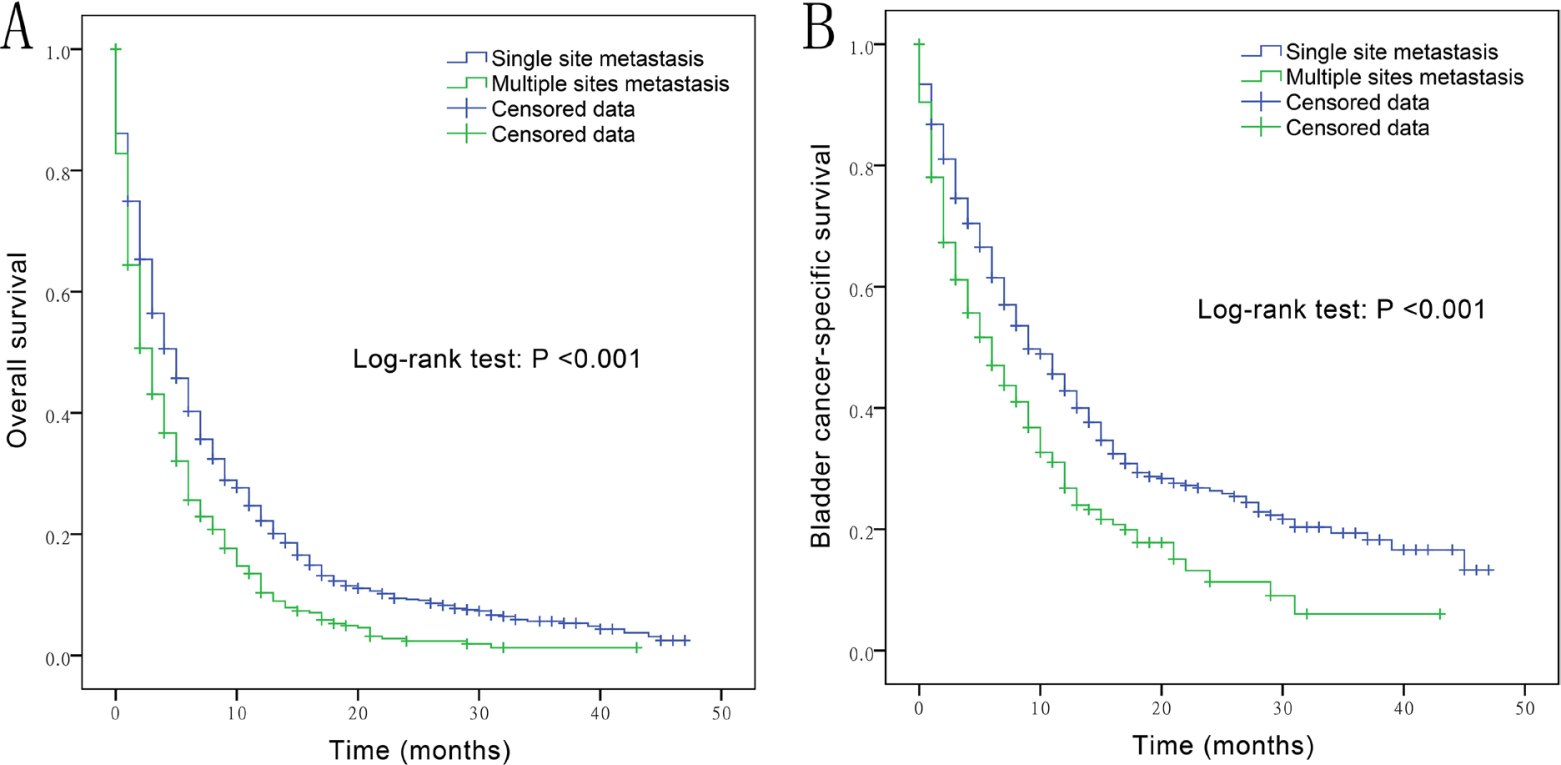
Overall survival (**A**) and bladder cancer-specific survival (**B**) in bladder cancer patients with single metastatic site *vs*. multiple metastatic sites.

**Figure 3:**
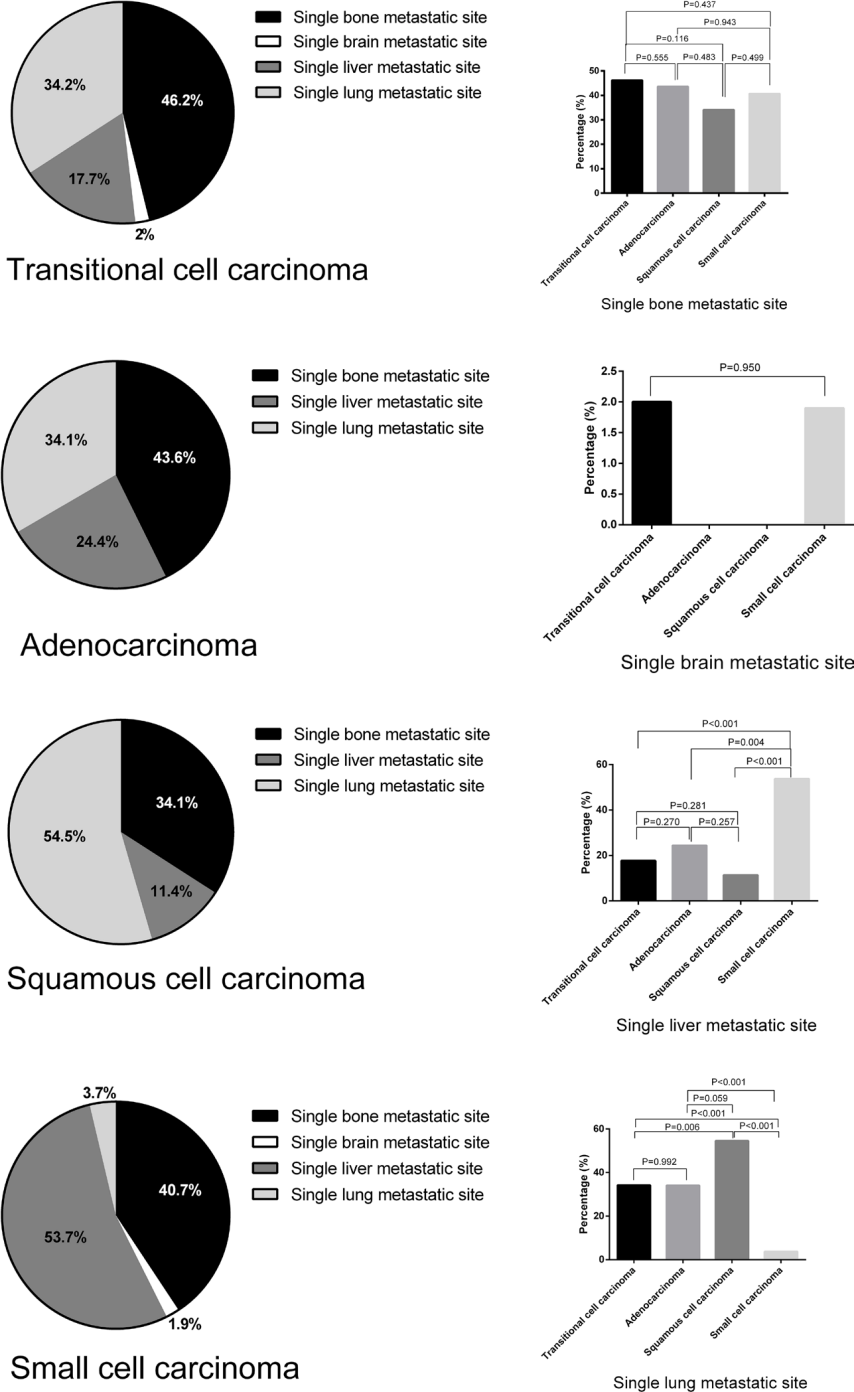
Metastatic frequency in patients with single-site metastatic bladder cancer by histological subtype

The median OS and CSS in patients with a single metastatic site was 5 and 9 months (range, 0–47), respectively, while that in patients with more than one metastatic site was 3 and 6 months (range, 0–43), respectively. As shown in Figure 2, the median OS and CSS of patients with multiple metastatic sites was significantly lower than that of patients with a single metastatic site (*P* < 0.001). Among patients with a single metastatic lesion, the frequency of bone and brain metastasis was almost the same. Lung was the most common site of metastasis in patients with SQCC (54.5%), while it was the least common metastatic site (3.7%) in those with SCC. Liver was the most frequent metastatic site in patients with SCC (53.7%). No brain metastasis was found in patients with AD and SQCC. As shown in Figure 3, a significant difference with respect to liver and lung metastasis was observed between SCC and other histological types (*P* ≤ 0.004), while a significant difference in lung metastasis was observed between SQCC with TCC and SCC (*P* < 0.006).

On univariate analysis (Table 2), thirteen variables showed a significant association with OS; these included age (> 65 years old), being widowed, pathological T (T1, T2, T3, T4) and N (N1, N2, N3) stages, history of surgery, presence of multiple metastatic sites, and AD and SQCC histological subtypes. Five variables showed a significant association with CSS (married status, history of surgery, presence of multiple metastatic sites, AD and SQCC histological subtypes). Four variables showed a significant association with both OS and CSS (history of surgery, presence of multiple metastatic sites, AD and SQCC histological subtypes). About 67.7% of all patients had undergone a surgical procedure, which included transurethral resection and partial, simple or radical cystectomy. As 2440 patients (92.6%) with metastatic sites were diagnosed at autopsy, only 78 (3%) patients had undergone surgery at the site of distant metastases. Significantly decreased hazard for OS and CSS was observed in patients who underwent surgery as compared to that in patients who did not undergo surgery (Hazard ratio [HR]: 0.660, 0.829, respectively). Presence of multiple metastatic sites was associated with lower OS and CSS (HR: 1.423, 1.537, respectively) than that associated with single site metastasis. Specifically, AD was associated with longer OS and CSS (HR: 0.697, 0.567, respectively) than that associated with the referent TCC. In contrast, SQCC was associated with shorter OS and CSS (HR: 1.504, 1.739, respectively) as compared to TCC.

**Table 2:** Univariate analysis of overall survival and bladder cancer-specific survival in patients with metastatic bladder cancer

Variables	Overall survival			Bladder cancer-specific survival		
	Hazard Ratio	95% CI	*P* value	Hazard Ratio	95% CI	*P* value
Age						
≤ 65	1.000 (reference)			1.000 (reference)		
> 65	1.339	1.220 to 1.469	**< 0.001**	1.038	0.922 to 1.168	0.540
Gender						
male	1.000(reference)			1.000 (reference)		
female	0.917	0.836 to 1.006	0.066	0.909	0.804 to 1.027	0.127
Race						
White	1.000 (reference)			1.000 (reference)		
Black	1.076	0.935 to 1.240	0.307	0.995	0.820 to 1.207	0.960
Other	0.936	0.767 to 1.142	0.514	0.864	0.657 to 1.135	0.294
Marital status						
Single	1.000 (reference)			1.000 (reference)		
Married	0.885	0.783 to 1.001	0.052	0.796	0.680 to 0.933	**0.005**
Divorced/Separated	0.990	0.845 to 1.160	0.905	0.931	0.759 to 1.142	0.491
Widowed	1.166	1.010 to 1.345	**0.036**	0.967	0.799 to 1.169	0.727
T stage						
T0 + Tx	1.000 (reference)			1.000 (reference)		
T1	0.758	0.657 to 0.875	**< 0.001**	0.960	0.787 to 1.172	0.690
T2	0.722	0.644 to 0.810	**< 0.001**	0.985	0.839 to 1.157	0.855
T3	0.667	0.562 to 0.790	**< 0.001**	0.938	0.750 to 1.174	0.578
T4	0.847	0.743 to 0.967	**0.014**	1.043	0.866 to 1.256	0.656
N stage						
N0 + Nx	1.000 (reference)			1.000 (reference)		
N1	0.801	0.685 to 0.937	**0.005**	0.913	0.747 to 1.115	0.371
N2	0.853	0.759 to 0.959	**0.008**	0.958	0.824 to 1.114	0.579
N3	0.753	0.617 to 0.919	**0.005**	0.869	0.675 to 1.119	0.277
Grade						
G1	1.000 (reference)			1.000 (reference)		
G2	1.019	0.595 to 1.745	0.945	0.944	0.443 to 2.011	0.880
G3	1.246	0.769 to 2.019	0.372	1.033	0.515 to 2.072	0.928
G4	1.088	0.673 to 1.758	0.731	0.875	0.435 to 1.758	0.708
Surgery						
No	1.000 (reference)			1.000 (reference)		
Yes	0.660	0.603 to 0.722	**< 0.001**	0.829	0.732 to 0.939	0.003
Radiotherapy						
No	1.000 (reference)			1.000 (reference)		
Yes	0.918	0.827 to 1.018	0.105	0.919	0.801 to 1.055	0.233
Distant metastasis						
Single site	1.000 (reference)			1.000 (reference)		
Multiple sites	1.423	1.275 to 1.588	**< 0.001**	1.537	1.331 to 1.774	< 0.001
Histology						
Transitional cell carcinoma	1.000 (reference)			1.000 (reference)		
Adenocarcinoma	0.697	0.540 to 0.889	**0.005**	0.567	0.391 to 0.821	**0.003**
Squamous cell carcinoma	1.504	1.216 to 1.862	**< 0.001**	1.739	1.335 to 2.265	**< 0.001**
Small cell carcinoma	0.985	0.800 to 1.213	0.888	0.946	0.715 to 1.251	0.696

Variables significantly associated with survival in univariate analysis were included in the multivariate analysis (Table 3). The effect of presence of multiple metastatic sites was further increased after accounting for other covariates (HRs for OS and CSS: 1.428 and 1.605, respectively, versus presence of single metastatic site). Compared with TCC, a lower hazard for OS and CSS was identified in patients with AD (HR: 0.593, 0.599, respectively), while a higher hazard of mortality was found in those with SQCC (HR: 1.496, 1.733 respectively). Other variables significantly associated with OS on multivariate analysis included age (> 65 years old) and history of surgery. Age > 65 years was associated with shorter OS (HR: 1.273 *vs*. age < 65 years). Patients who had a history of surgery tended to have longer OS (HR: 0.729 *vs*. those with no history of surgery).

**Table 3:** Multivariate analysis of overall survival and bladder cancer-specific survival in patients with metastatic bladder cancer

Variables	Overall survival			Bladder cancer-specific survival		
	Hazard Ratio	95% CI	*P* value	Hazard Ratio	95% CI	*P* value
Age						
≤ 65	1.000 (reference)					
> 65	1.273	1.116 to 1.452	< 0.001			
Marital status						
Single	1.000 (reference)			1.000 (reference)		
Married	0.875	0.744 to 1.029	0.107	0.883	0.723 to 1.079	0.223
Divorced/Separated	1.027	0.836 to 1.262	0.799	1.044	0.806 to 1.352	0.744
Widowed	1.000	0.820 to 1.219	0.999	0.989	0.775 to 1.262	0.928
T stage						
T0 + Tx	1.000 (reference)					
T1	0.984	0.802 to 1.207	0.875			
T2	1.000	0.829 to 1.206	1.000			
T3	0.944	0.731 to 1.218	0.657			
T4	1.028	0.838 to 1.262	0.788			
N stage						
N0 + Nx	1.000 (reference)					
N1	0.877	0.712 to 1.080	0.215			
N2	0.942	0.800 to 1.109	0.475			
N3	1.148	0.857 to1.537	0.355			
Surgery						
No	1.000 (reference)			1.000 (reference)		
Yes	0.729	0.630 to 0.845	**< 0.001**	0.975	0.817 to 1.165	0.781
Distant metastasis						
Single site	1.000 (reference)			1.000 (reference)		
Multiple sites	1.428	1.260 to 1.618	**< 0.001**	1.605	1.367 to 1.885	**< 0.001**
Histology						
Transitional cell carcinoma	1.000 (reference)			1.000 (reference)		
Adenocarcinoma	0.593	0.427 to 0.825	**0.002**	0.599	0.389 to 0.922	**0.020**
Squamous cell carcinoma	1.496	1.121 to 1.995	**0.006**	1.733	1.225 to 2.452	**0.002**
Small cell carcinoma	0.860	0.678 to 1.091	0.213	0.782	0.568 to 1.078	0.134

## DISCUSSION

We conducted a retrospective cohort study using data from the SEER database to investigate the effect of histological sub-type on survival in patients with metastatic BC. Our study revealed several important findings. First, although the association between histological subtype and prognosis of BC has been investigated before, previous population-based studies were limited only to one rare histological subtype of BC such as SQCC [7–9], AD [18, 19] or SCC [4, 5, 20–24], or had relatively small sample sizes [6]. SQCC histology was reported to be an independent predictor of higher all-cause and bladder cancer-specific mortality as compared to TCC among patients with advanced stages (AJCC stage III/IV) [7]. However, some studies found no difference in CSS associated with SQCC and TCC [6, 8, 9, 25]. A large cohort study showed that patients with primary AD were not associated with worse prognosis than patients with TCC [26]. We found that AD patients had better survival outcomes than those with conventional TCC, while SQCC had worse outcomes than TCC. Further, there were no significant differences in OS and CSS between TCC and SCC patients in stage M1 group, which is in accordance with results of a previous study [27].

Secondly; we discovered a difference in metastatic tendency between the SQCC and SCC subtypes of BC. We found that SQCC metastasizes predominantly to lung (54.5%), while SCC metastasizes predominantly to liver (53.7%) and is least likely to metastasize to lung (3.7%) when compared with other histological subtypes of BC. These results are in line with studies conducted at the M. D. Anderson Cancer Center [28] and Mayo Clinic [29]. Although brain metastases are a frequent complication in patients with SCC of the lung, they do not appear to be a common metastatic site in patients with SCC of the bladder [20, 22, 30]. We also found only one patient (1.9%) who developed brain metastases. Due to the low incidence of brain metastases in SCC of the bladder, prophylactic cranial irradiation is not routinely recommended. In cases of metastatic BC, transurethral resection, cystectomy combined with systemic chemotherapy has been proposed apart from partial cystectomy, and radiotherapy [22]. However, there is a paucity of robust data to inform appropriate tailored therapies for different histological subtypes of metastatic BC.

Limitations of our study include the retrospective design and its associated bias, such as selection bias for different treatment schedules. Moreover, due to the inherent limitations of the SEER database, data on specific metastatic sites prior to year 2010 were not available; therefore, the reference period for this study was 2010–2013. We also have no access to data on performance status and chemotherapy which are also important prognostic factors for patients with metastatic BC. So the use of claims based data for this approach will lead to somewhat insufficient estimations. Despite the above limitations, our study fills a gap with respect to histological subtypes and metastatic sites of BC.

In conclusion, based on the SEER database, we compared survival outcomes by histological subtype and site of metastatic lesions in patients with bladder cancer. Our findings suggest that squamous cell carcinoma and presence of multiple metastases portend a poorer prognosis in bladder cancer patients with distant metastasis. Obvious associations are reported with respect to metastatic frequency among patients with squamous and small cell carcinoma. Considering the poor prognosis of metastatic bladder cancer, we should keep an eye on those susceptible organs and sites once the histological subtype is determined.

## MATERIALS AND METHODS

### Study design and population

We identified all patients from 2010–2013 in the Surveillance, Epidemiology, and End Results (SEER) database with BC diagnosed as stage M1, according to the AJCC (American Joint Committee on Cancer) 7th edition. The permission to access the research data was obtained vide reference number 10263-Nov2015. This study was approved by the Clinical investigation ethics committee of the Soochow University (No. 2015103). We used International Classification of Disease for Oncology, Third Edition (ICD-O-3) to determine the histological sub-type. The codes selected were: transitional cell carcinoma (8120, 8122, 8130 and 8131); adenocarcinoma (8140, 8574, 8260 and 8255); squamous cell carcinoma (8052, 8070, 8071 and 8083); and small cell carcinoma (8041 and 8045).

### Outcomes

Overall survival (OS) was defined as the time period from the first diagnosis of bladder cancer to the date of all-cause death, while bladder cancer-specific survival (CSS) was determined until cancer-specific death or censoring, with the end of follow-up occurring on December 31, 2013.

### Covariates

Demographic characteristics included age (< 65 and > 65 years old), gender (male and female), race (white, black and others), and marital status (single, married, divorced/separated and widowed). Clinical characteristics included pathological T and N stages, tumor grade (1-well differentiated, 2-moderately differentiated, 3-poorly differentiated and 4-undifferentiated), surgery, radiotherapy, distant metastasis (single site and multiple sites) as well as tumor histology (TCC, AD, SQCC and SCC).

### Statistical analysis

Categorical variables of Table 1 and metastatic rates of Figure 3 are presented as percentages and analyzed by Chi-squared test and Fisher's exact test. In Figure 1 and Figure 2, the Kaplan-Meier method was used to calculate sur*vivo*r functions and differences in OS, CSS probabilities were estimated using the log rank statistic. The Cox proportional hazards model was used to assess association of variables with survival. Variables showing a significant association with survival on univariate analysis were included in the multivariate model. Statistical analyses were performed using SPSS version 21 (IBM, Chicago, IL, USA); two-sided value of P<0.05 was considered statistically significant. The bar and pie charts were drawn using GraphPad Prism version 6.01 (San Diego, CA).

## Abbreviations

(BC): Bladder Cancer
(OS): Overall Survival
(CSS): Bladder cancer-specific survival
(HR): Hazard ratio
(TCC): Transitional cell carcinoma
(SQCC): Squamous cell carcinoma
(AD): Adenocarcinoma
(SCC): Small cell carcinoma
(SEER): Surveillance, Epidemiology, and End Results
(AJCC): American Joint Committee on Cancer
(ICD-O-3): International Classification of Disease for Oncology, Third Edition
